# Editorial: Digital therapeutics: using software to treat, manage, and prevent disease

**DOI:** 10.3389/fdgth.2023.1261124

**Published:** 2023-09-25

**Authors:** Kirsten E. Smayda, Tim Campellone, Sabrina R. Taylor, Brian Harris, Terry D. Ellis, Louis N. Awad

**Affiliations:** ^1^ MedRhythms, Portland, ME, United States; ^2^ Woebot Labs Inc., San Francisco, CA, United States; ^3^ College of Health and Rehabilitation Sciences, Sargent College, Boston University, Boston, MA, United States

**Keywords:** DTx, digital therapeutics, software, hardware, medical device, intervention, symptom monitoring, clinical tool

**Editorial on the Research Topic** Digital therapeutics: using software to treat, manage, and prevent disease

The Research Topic, *Digital Therapeutics: Using Software to Treat, Manage, and Prevent Disease*, was developed to build a collection of evidence that exhibits and incites innovation of novel digital therapeutics (DTx) and their component technologies, and to showcase outcomes that meet the needs of all stakeholders in the process of commercializing digital therapeutics. This Topic and Editorial primarily focuses on the U.S.A. market, although two articles are from teams in Canada, Germany, and Switzerland, and is particularly timely given the state of technological progress and the digital therapeutics industry.

We live in a time when digital technology has advanced such that interventions can be delivered in a clinically meaningful way. Technology can now support high-fidelity data collection (e.g., biometric, physiological, and kinematic), appropriate controls, feedback loops, and detailed visual and auditory resolution—all to support the delivery of an intervention. Macrotrends in healthcare also make it an ideal moment for this Research Topic: an increasing momentum and focus on reimbursement of DTx, and the recent rise of telehealth due to the COVID-19 pandemic. While this Topic focuses on DTx, defined by ISO/TR 11147:2023(en) and the DTx Alliance as “…health software intended to treat or alleviate a disease, disorder, condition, or injury by generating and delivering a medical intervention that has a demonstrable positive therapeutic impact on a patient's health,” (1, 2) other products and services within the digital health ecosystem, including patient symptom monitoring and clinical support tools, are included in this topic and represent the landscape within which DTx exist.

This Research Topic highlights work from commercial, academic, and collaborations across both entities and includes a range of clinical and care populations including depression, multiple sclerosis, opioid-based pain management, stroke, language, cognition, dementia, and attention-deficit/hyperactivity disorder. Amongst the articles that describe results of an intervention study, Kulikov et al. addresses the gap between need and access to evidence-based services for adolescent mental health by presenting initial, positive, evidence from a randomized controlled trial on the feasibility and acceptability of a digital therapeutic, Spark, to treat depression in adolescents. Cuyler et al. finds that a 28-day home-based Capnometry Guided Respiratory Intervention could support symptom reduction and adherence in people with panic disorder and post-traumatic stress disorder. DTx also offer an unprecedented view into engagement patterns during treatment. Heusser et al. describes a machine learning model that measures quality of user interactions and intended use, and provides a helpful contextual framing for DTx in the introduction, as well. Liu et al. characterizes the relationship between engagement/dosage and improvements across 13 skill domains in people who had a stroke that resulted in speech, language, and cognitive deficits, finding that a higher dosage is related to greater improvement in in-home therapy outcomes over 6 months.

Two articles provide original research related to tracking symptoms and outcomes in real-time. Chen et al. sought to “identify divergent factors that influence subjectively and objectively measured cognitive functioning in real time in people with multiple sclerosis” and DiCarlo et al. finds that “SMS texting is a feasible method for gathering outcomes after stroke at scale to evaluate the efficacy of acute stroke treatments.” Two articles focus on supporting the care team of patients. Melvin et al. provides a “common nomenclature” to be used by clinicians and developers to support interpretation and application of artificial intelligence models. And Braun et al. describes PHREND®, an algorithm updated with new data and can “predict freedom of relapse and 3-months confirmed disability progression” to support decision-making between patient and clinician.

Two articles provide forward-looking perspectives. In Watson et al., the authors elucidate the gaps within the evolving and dynamic regulatory landscape for how prescription digital therapeutics (PDTs) are currently evaluated for safety and efficacy and regulated by the U.S. Food and Drug Administration (FDA). Russo et al. presents a “theoretical background, rationale, and development plans” for a music-based digital therapeutic to manage agitation and anxiety in people with dementia.

Lastly, one article, Giravi et al., reviews the literature on the “clinical evidence of digital interventions delivered via virtual reality and mobile apps to improve opioid-based analgesia” and concluded that they can improve pain scores compared to “treatment as usual”.

The editorial team experienced two learnings, in particular, that might benefit the digital therapeutics industry to consider. First, Institutional Review Boards (IRBs) are important partners in conducting research in both non-commercial and commercial contexts. If a company or research group intends to publish data from commercial users of a product or service, it is important that the researchers still submit their protocol to an IRB for exempt status determination under 45 CFR § 46.104(d)(4), if using de-identified data. This is ideally done prospectively before the data is collected, but can also be done retroactively. Having an exemption determination in-hand can help expedite the review process, and could also be complemented by clear language in a privacy notice or terms of service informing the user that their data may be used for research and publication purposes.

Second, this Research Topic is missing research that includes payers and implementation studies. In the future, we encourage researchers to consider working with payers because of the critical role they play in reimbursing digital therapeutics, and also conducting implementation research to identify and plan for barriers to successful adoption. There were, however, several articles that represented collaborations across types of institutions. For example, the team from Pear Therapeutics co-authored their manuscript with the Devices division at Sanofi, a global pharmaceutical company (Watson et al.); the team at Freespira worked with the Laboratory for the Study of Anxiety Disorders at The University of Texas at Austin (Cuyler et al.); Chen et al. involved a collaboration across Rutgers, University of Illinois, and the Kessler Foundation; and Russo et al., involved a collaboration across multiple universities in Toronto, LUCID Inc., and Right to Music. These collaborations paint an evolving picture of the collaborations needed to bring evidence-based digital therapeutics that are rooted in good science and the reality of bringing DTx to market.

In conclusion, the editorial team is grateful to everyone who submitted their article for consideration in this important Research Topic. The quality and breadth of research in this Topic bolsters the foundation of evidence for not only the products and services represented in this Research Topic, but also the digital therapeutics industry at large. It is the editorial team's earnest hope that the work presented here will add to the momentum for digital therapeutics to be adopted by the many stakeholders in healthcare, including providers, payers, and patients.
